# The Burden of Early Childhood Caries in Canadian Children and Associated Risk Factors

**DOI:** 10.3389/fpubh.2019.00328

**Published:** 2019-11-12

**Authors:** Andrew Pierce, Sarbjeet Singh, JuHae Lee, Cameron Grant, Vivianne Cruz de Jesus, Robert J. Schroth

**Affiliations:** ^1^Dr. Gerald Niznick College of Dentistry, Rady Faculty of Health Sciences, University of Manitoba, Winnipeg, MB, Canada; ^2^Children's Hospital Research Institute of Manitoba, Winnipeg, MB, Canada; ^3^Maxy Rady College of Medicine, Rady Faculty of Health Sciences, University of Manitoba, Winnipeg, MB, Canada

**Keywords:** early childhood caries (ECC), Canada, preschool child, risk factors, nutritional status, burden of caries

## Abstract

**Introduction:** Early childhood caries (ECC) is any caries in the primary dentition occurring in children under the age of six. ECC is common in many population groups in Canada.

**Objective:** The purpose of this review was to describe the burden of ECC in Canada, the prevalence and associated risk factors for ECC, and its impact on childhood health based on the existing published literature.

**Methods:** A review was conducted to assess published Canadian studies on ECC identified through searches of electronic databases. Databased searched included PubMed, Medline, Cinahl, and the library catalog of the University of Manitoba. Known publications on ECC that were not identified by the electronic search were also considered. Only the studies that reported the prevalence of ECC or caries in preschool aged children were considered. In-depth assessments were restricted to those studies that employed logistic regression analysis to investigate relationship between ECC and risk factors or nutritional status and quality of life.

**Results:** A total of 36 studies were identified that related to ECC in Canadian children. Overall, 27 related to prevalence and 12 reported on risk factors, four related to the association between severe ECC and nutritional health and well-being, while only one related to the oral microbiome composition. Published studies reveal that the prevalence of ECC can be as high as 98% in some parts of Canada. Commonly identified risk factors include age, sex, socio-economic status, parental beliefs, family characteristics, debris/plaque, enamel hypoplasia, and behavioral (oral health or feeding behaviors) tendencies.

**Conclusions:** Current literature reveals that many Canadian children are affected by ECC. The development of ECC appears to be strongly associated with social determinants of health including low household income and the level of parental education or employment status. Associations were also observed between ECC and the child's age at first dental visit and parental beliefs about child's oral health. Children with enamel hypoplasia are also at significantly greater odds for experiencing caries. Future research should include assessments of developmental defects of enamel to better understand the association between enamel hypoplasia and ECC.

## Introduction

Decay involving the primary dentition in children younger than 6 years of age is termed Early Childhood Caries (ECC) (1). ECC is a multifactorial chronic disease, which is influenced by biomedical factors such as diet, the oral microbiome, tooth integrity and by underlying social determinants of health (1, 2). This includes low socioeconomic status (SES), parental education, maternal nutrition, and psychosocial issues (3–5).

Numerous young Canadian children experience significant oral health problems, particularly ECC. This is especially true for First Nations, Inuit and Metis children, recent refugees and immigrants (i.e., newcomers), those living in low SES households, and children from geographically remote regions of the country (6–12). It is now well-established that up to 90% of children in some northern and remote Indigenous communities are affected (13–15). Usually, urban children are considered low-risk for caries, but there is also data available that signify that ECC is prevalent in disadvantaged urban communities in Canada (11, 16, 17). The challenge is that those most at risk for ECC are often those with the greatest barriers in accessing oral health care services.

Although national data on the true prevalence of ECC is missing, as children <6 years of age were not included in the Canadian Health Measures Survey, ECC continues to be a problem in Canada today (3, 18). One reason is due to the failure of previous interventions of being able to address successfully the underlying causative biomedical, behavioral, and social factors known to contribute to the development of caries (12).

If left untreated, early childhood caries advances to a more rampant form of disease, which further leads to malocclusions, abscess and pain. This situation is further compounded by the fact that there are long waiting lists for operative treatment in hospitals (19, 20). Furthermore, Severe Early Childhood Caries (S-ECC) among young children can negatively impact oral health related quality of life, nutritional status and growth (12, 21–23). There is also a growing body of evidence that the reoccurrence of dental caries and relapse following restorative dental treatment under general anesthesia is high (24–26).

In the absence of national prevalence data, national rates of pediatric dental surgery to treat ECC under general anesthesia can serve as a proxy measure (3, 27). National data reveals that the rate of dental surgery for ECC is 12.1 per 1,000 children and is recognized as the most common surgical procedure performed in preschool children at most Canadian hospitals (3, 28). This report also found that children from rural regions had rates of dental surgery to treat ECC 3.2 times higher than rates of urban-dwelling children (31.2 per 1,000 vs. 9.8 per 1,000) (3).

Due to potential for ECC to affect childhood growth and development and the associated treatment costs, the prevalence and etiology of ECC must be examined more closely to inform clinical management, policy, and prevention activities. The purpose of this review was to describe the burden of ECC in Canada, particularly its prevalence, associated risk factors, and its impact on childhood health and well-being based on the existing literature.

## Methods

Electronic databases were searched to identify all Canadian published studies on ECC. The search strategy by Schroth and Grant informed our work for this current review and provided a recent listing of relevant publications (18). Keywords (used alone and in various combinations) were: baby, babies, Canada, Canad^*^, caries, cavities, cavit^*^, child, “dental caries,” “dent^*^ and cavit^*^,” “early childhood caries,” ECC, infant, preschool^*^, “severe early childhood caries,” S-ECC, and toddler. The search terms were used for title, abstract, and where possible full text. As the term and case definition for early childhood caries was adopted in 1998 (29), the literature search was limited to sources published in the English language spanning from 1990 to 2019. Databases searched included PubMed, Medline, Cinahl, Scopus, Cochrane Library, and the Library Catalog of the University of Manitoba. Similar search strategies were used to identify web resources using the Google search engine. Known publications on ECC that were not identified by the electronic search were also considered.

For the purpose of this review, ECC was defined according to American Academy of Pediatric Dentistry's case definition (1). Studies were included if they involved children <72 months of age, involved Canadian children and either focused on ECC, reported the prevalence of caries in the primary dentition (i.e., ECC, dmft > 0, dmfs >0), examined the risk factors for ECC, investigated the associated microbiome with modern techniques, or discussed associations between ECC and nutritional status and well-being. Studies relating to parental reported ECC were not included (27, 30). In depth assessments were restricted to those studies that employed logistic regression analysis to investigate the relationship between ECC and risk factors or nutritional status and quality of life. Studies exploring the oral microbiome were also reviewed in detail.

Common risk factors for ECC investigated by Canadian studies were identified with variables grouped into 15 overarching categories: childhood age, sex, SES, community of residence, family characteristics, ethnicity, parental beliefs, prenatal nutrition, child behaviors, feeding behaviors (e.g., bottle-feeding and breastfeeding), dental history, enamel hypoplasia, tooth debris score, fluoride exposure, and oral health behaviors (e.g., brushing habits). SES risk factors included any demographic characteristics relating to the level of parental education and annual household income. Family characteristics encompassed variables related to the family size or composition and whether the caregiver had legal custody of the child.

## Results

A total of 36 published Canadian studies were identified and reviewed in detail; 27 reporting prevalence, 11 reporting associated risk factors following logistic regression analyses, six exploring associations with nutrition and well-being, and one involving the oral microbiome (Table 1). Twenty-one of the studies reviewed were cross-sectional in nature, seven were case-control studies, three were randomized control trials, three were retrospective chart reviews, and two studies employed a prospective cohort design. A total of 14 studies involved Indigenous children (i.e., First Nations, Inuit, or Metis), six included children of newcomers (immigrants and refugees) to Canada, and 11 were with ethnically diverse populations.

**Table 1:** Published studies on early childhood caries among pre-school children in Canada over 29 years.

**Study**	**Region of Canada**	**Population**	**Type of study**	**Age**	**Prevalence of ECC (%)**	**Reported risk factors using multiple logistic regression**
Williams and Hargreaves (31)	Edmonton, Alberta	Urban Asians	Cross-sectional	2 years old 3–4 years old 5 years old	0 28 62	No
Harrison and Davis (32)	British Columbia (1988 Survey)	First Nation	Cross-sectional	5 Years old	87.5 in 1988 89.7 in 1980	No
Young et al. (33)	Keewatin region, Northwest Territories	Inuit	Cross-sectional	0–2 years old 3-5 years old	50–100, depending on community	No
Weinstein et al. (34)	Edmonton, Alberta	Urban Caucasian and diverse ethnic	Cross-sectional	Mean age = 19 months	25	No
Harrison et al. (35)	Vancouver, British Columbia	Urban Vietnamese	Cross-sectional	3–74 months <18 months ≥18 months	0 79.5	No
Harrison and Wong (17)	Vancouver, British Columbia (1996 follow-up)	Urban Vietnamese (Intervention Group Control group)	Cross-sectional and intervention study	22.1 ± 5.0 months 22.7 ± 5.8 months	6.2 intervention 57.1 comparison	No
Peressini et al. (36)	District of Manitoulin, Ontario	First Nation	Cross-sectional	3 years old 5 years old	67 78	No
Lawrence et al. (14)	Northern Ontario	First Nation	Cross-sectional	2 years old 3 years old 4 years old	91.8 S-ECC-−81.9 97.7 S-ECC−95.8 100 S-ECC−94.7	No
Schroth and Moffatt (13)	Garden Hill First Nation, Manitoba	First Nation	Cross-sectional	3–5 years old	98.9	No, but did undertake multiple regression for deft score
Schroth et al. (10)	Rural South-western Manitoba	Caucasian	Cross-sectional	36–48 months	44 S-ECC−21	Yes
Schroth et al. (16)	Winnipeg (South Point Douglas), Thompson, Norther First Nation, Roseau River First Nation, Manitoba	Diverse urban and First Nations children living on reserve	Cross-sectional	<1–5 years old <1 year old 1 year old 2 years old 3 years old 4 years old 5 years old Winnipeg (South Point Douglas) Thompson Northern First Nation Roseau River First Nation	53.6 7.1 30.4 54.7 78.1 78.3 84.2 43.3 51.4 58.6 56.5	No
Clarke et al. (21)	Toronto, Ontario	Ethnically heterogeneous children with S-ECC	Cross-sectional	2–6 years of age	N/A All children recruited had S-ECC.	No
Harrison et al. (37)	Hartley Bay, British Columbia	First Nations (Base Line Group)	Cross-sectional	Pre-kindergarten children	69 (baseline)	No
Schroth et al. (38)	Winnipeg (South Point Douglas), Thompson, Norther First Nation, Roseau River First Nation, Manitoba	Diverse urban and First Nations children living on reserve	Cross-sectional	<72 months of age	Previously reported in Schroth et al. (16)	Yes
Harrison et al. (39)	Surrey, British Columbia	South Asian immigrants enrolled into a randomized trial of motivational interviewing	Randomized controlled trial (RCT)	6–18 months (baseline) Intervention group Control group	1.6	No, but did undertake Poisson regression for dmfs
Schroth and Cheba (40)	Winnipeg and surrounding area in southern Manitoba	Ethnically heterogeneous	Retro-spective chart review	1-5 years old <23 months 2 years old 3 years old 4 years old 5 years old	71 47 56 67 75 78	Yes
Tiberia et al. (41)	Five Pediatric dental practices in Canada—British Columbia, Ontario, Prince Edward Island	77% Caucasian, 7% First Nation, 8% Asian	Cross-sectional survey	Mean age 3.05 years 1–5 year olds 1–2 year olds	74.5 S-ECC−65.7 72.7 S-ECC−57.6	Yes
Leake et al. (42)	Inuvik Region, Northwest Territories	Predominantly Inuit	Cross-sectional	2–5 years old 2 years old 3 years old 4 years old 5 years old	62.5 29 53 67 69	Yes, but this was undertaken for their own case definition for S-ECC
Valencia-Rojas et al. (43)	Toronto, Ontario	Children in care of the Children's Aid Society of Toronto (Maltreated Children)	Retro-spective chart review	2–6 years old 2–3 years old 4–6 years old	57.6 50.0 63.9	Yes
Werneck et al. (44)	Toronto, Ontario	Portuguese speaking immigrants from Portugal or the Azores, Brazil, Angola, or Mozambique	Case-control	≤ 48 months of age	35	Yes
Lawrence et al. (45)	Sioux Lookout Zone (SLZ) and Thunder Bay, Ontario	First Nations children in SLZ and primarily non-Indigenous comparison group in Thunder Bay	RCT	6 months to 5 years old Baseline: SLZ control SLZ fluoride varnish intervention Thunder Bay intervention	68.9 72.7 15.7	Yes
Lawrence et al. (46)	Sioux Lookout Zone (SLZ) and Thunder Bay, Ontario	First Nation Primarily non-aboriginal	Cross-sectional	<6 years of age SLZ 3–5 year olds Thunder Bay Kindergarten All Non-Indigenous living off-reserve	95.9 (2001) 94.6 (2002) 70.6 (2003) 89.1 (2004) 93.8 (2005) 37.7 (2003/04) 40.3 (2004/05) 36.8 (2005/06) 31.1 (2003/04) 35.2 (2004/05) 31.2 (2005/06) 73.8 (2003/04) 81.6 (2004/05) 79.4 (2005/06)	No, but did undertake Poisson regression
Schroth et al. (47)	South-western Manitoba	Hutterite Colonies	Cross-sectional	6–71 months	53 S-ECC– 42.4	Yes
Harrison et al. (48)	Nine Cree communities in northern Quebec	First Nations	RCT	Children of mothers who had recently given birth	Control group 76% (100/131) Treatment group 65% (72/110) Overall, 0.71	
Schroth et al. (49)	Winnipeg and surrounding southern Manitoba	Ethnically Heterogeneous	Case-control	Mean age 43.8 months 16–71 months of age	N/A Children were recruited based on whether or not they had S-ECC	Yes
Schroth et al. (23)	Winnipeg, Manitoba	Ethnically Heterogeneous	Case-control	Mean age 40.8 <72 months of age	N/A Children were recruited based on whether or not they had S-ECC	Yes
Schroth et al. (22)	Winnipeg, Manitoba	Ethnically Heterogeneous	Case-control	Mean age 40.8 <72 months of age	N/A Children were recruited based on whether or not they had S-ECC	Yes
Schroth et al. (50)	Winnipeg (93%) and surrounding rural Manitoba (7%)	Expectant mothers from vulnerable populations. 90% Canadian Aboriginal (First Nation, Metis, or Inuit)	Prospective cohort	Infants <24 months of age	23– when restricted to cavitated enamel lesions 36–when white spot lesions are included S-ECC-−23	Yes
Schroth et al. (51)	Winnipeg (South Point Douglas), Thompson, Northern First Nation, Roseau River First Nation, Manitoba	Cross-sectional follow-up study evaluating the Healthy Smile Happy Child Initiative	Cross-sectional	Children ≤ 71 months of age	52 S-ECC-−38.6	Yes
Amin et al. (9)	Edmonton, Alberta	African Communities (90% originated from North eastern Africa; 9.7% from West Africa)	Cross-sectional	21–72 months old	63.7 (untreated ECC)	No
Poon et al. (52)	British Columbia (Province Wise)	Kindergarten children 4–6 years of age	Retro-spective cohort	Children between the ages of 4 and 6 years old	38.9 (2006/07) 36.7 (2009/10)	No
Davidson et al. (53)	Winnipeg, Manitoba	Ethnically heterogeneous	Case-control	24–71 months old	N/A Children were recruited based on whether or not they had S-ECC	No, but did undertake multiple linear regression for BMI that included S-ECC as a covariate
El Azrak et al. (11)	Winnipeg, Manitoba	Children from Newcomer families	Cross-sectional	<72 months of age	45.5 S-ECC−31.3	Yes
Agnello et al. (54)	Winnipeg, Manitoba	First Nation or Metis.	Case-control	<72 months of age	N/A Children were recruited based on whether or not they had S-ECC to look at the microbiome	No
Deane et al. (55)	Winnipeg, Manitoba	Ethnically heterogeneous	Case-control	40.8 † 14.1 months	N/A Children were recruited based on whether or not they had S-ECC	No, but did undertake multiple linear regression for PTH levels that included S-ECC as a covariate
Grant et al. (56)	Winnipeg, Manitoba	Children with S-ECC receiving treatment under general anesthesia	Prospective cohort	≤ 6 years of age	N/A Children with S-ECC were specifically recruited.	No, but multiple linear regression undertaken for early childhood oral health impact scale scores

Among the entire number of studies reviewed, only 14 incorporated multiple logistic regression into their analysis to investigate the presence of any significant relationships between ECC and several associated variables (Table 2). Eleven of these studies focused their analysis on risk factors that may contribute to the onset of ECC, while three examined how severe forms of ECC are associated with underlying nutritional status of affected children.

**Table 2:** Identified risk factors for ECC in Canadian studies.

**Study**	**Risk factors for ECC or S-ECC from multiple logistic regression analyses**	**Association with nutrition and well-being**
Schroth and Moffatt (10)	**Not Significant:** Family size (*p* > 0.05) [family characteristics] Duration of breastfeeding (*p* > 0.05) [feeding behavior] Bottle at bed (*p* > 0.05) [feeding behavior] Gender (*p* > 0.05) [sex] Debris on teeth (*p* > 0.05) [debris]	
Schroth et al. (38)	**Significant:** Disagree that it is okay to let baby nurse in bed with mother all night (OR = 0.55, *p* = 0.018) [beliefs] Rotten teeth could affect child's health (OR = 4.33, *p* = 0.006) [beliefs] **Not Significant:** Bottle feeding after child is 1 year old is bad for his/her teeth (OR = 0.70, *p* = 0.162) [beliefs]	
Tiberia et al. (41)	**Significant:** Leaving bottle with child with ECC (RR = 6.71, *p* = 0.002) and S-ECC (RR = 7.43, *p* = 0.002) [feeding behavior] Having problems brushing with ECC (RR = 3.11, *p* = 0.05) [oral hygiene behavior] Holding liquids in the mouth for prolonged time with ECC (RR = 7.04, *p* = 0.04) [feeding behavior] Using a bottle [protective] with ECC (RR = 0.11, *p* = 0.01) and S-ECC (RR = 0.16, *p* = 0.05) [feeding behavior] Having a difficult child [protective] with ECC (RR = 0.08, *p* = 0.04) [behavior] Caucasian with S-ECC (RR = 7.22, *p* = 0.05) [ethnicity] **Not Significant:** Receive government assistance with ECC (RR = 2.17, *p* = 0.34) and S-ECC (RR = 2.18, *p* = 0.34) [SES] Having problems brushing with S-ECC (RR = 2.36, *p* = 0.17) [oral hygiene behavior] First born child with ECC (RR = 1.98, *p* = 0.21) and S-ECC (RR = 2.06, *p* = 0.22) [family characteristics] Having a difficult child [protective] with ECC (RR = 0.09, *p* = 0.12) [behavior] Holding liquids in the mouth for prolonged time with S-ECC (RR = 5.60, *p* = 0.07) [feeding behavior] Level of parental education with ECC (RR = 1.84, *p* = 0.34) and S-ECC (RR = 2.44, *p* = 0.21) [SES] Caucasian with ECC (RR = 3.11, *p* = 0.16) [ethnicity] Timing of snacks with ECC (RR = 1.65, *p* = 0.37) and S-ECC (RR = 1.80, *p* = 0.33) [feeding behavior] Fluoridated water with ECC (RR = 0.63, *p* = 0.22) and S-ECC (RR = 0.53, *p* = 0.11) [fluoride] Daily medications with ECC (RR = 4,385.24, *p* = 0.79) and S-ECC (RR = 3,307.67, *p* = 0.80) [health status]	
Schroth and Cheba (40)	**Significant:** Male child (OR = 1.38, *p* = 0.048) [sex] Age at first visit >23 months of age (OR = 0.19, *p =* 0.009) [age] Low monthly income (≤ $2000 per month) (OR = 1.79, *p =* 0.014) [SES] Failed dental appointments (OR = 1.82, *p =* 0.002) [dental history] Not being a single parent (OR = 0.61, *p =* 0.011) [family characteristics] **Not Significant:** Community of residence (urban) (OR = 0.55, *p =* 0.06) [community of residence] Low monthly income per family member (< $325) (OR = 1.28, *p =* 0.092) [SES] Maternal employment status (being employed) (OR = 1.42, *p =* 0.054) [SES]	
Valencia-Rojas et al. (43)	**Significant:** Having guardianship status [protective] with ECC (OR = 0.14, *p =* 0.03) [family characteristics] Number of times in care of the Children's Aid Society of Toronto [protective] with S-ECC (OR = 0.16, *p =* 0.03) [family characteristics] **Not Significant:** Age with ECC (OR = 1.52, *p =* 0.45) and S-ECC (OR = 1.18, *p =* 0.79) [age] Sex with ECC (OR = 0.54, *p =* 0.29) and S-ECC (OR = 0.84, *p =* 0.77) [Sex] Siblings in care of Children's Aid Society of Toronto with ECC (OR = 0.74, *p =* 0.65) and S-ECC (OR = 0.81, *p =* 0.75) [family characteristics] Type of Abuse with ECC (OR = 1.09, *p =* 0.90) and S-ECC (OR = 0.61, *p =* 0.48) Number of times in care of the Children's Aid Society of Toronto with ECC (OR = 0.42, *p =* 0.16) [family characteristics] Having guardianship status with S-ECC (OR = 0.22, *p =* 0.24) [family characteristics] Period of time under care of Children's Aid Society of Toronto with S-ECC (OR = 1.29, *p =* 0.73) [family characteristics]	
Lawrence et al. (45)	**Significant:** Control vs. fluoride varnish (FV) (Adjusted OR = 1.96, *p =* 0.027) [fluoride] Baseline dfs (≥5 vs. 0–4) (Adjusted OR = 4.88, *p =* < 0.001) [dental history] Age (0–2 years vs. 3–5 years) (Adjusted OR = 1.94, *p =* 0.02) [age] **Not Significant:** Sex (Female vs. Male) (Adjusted OR = 1.14, *p =* 0.307) [sex] Follow-up timing (18–24 months vs. 12–18 months) (Adjusted OR = 1.33, *p =* 0.187) [age] Parental Education (< High School vs. ≥High School) (Adjusted OR = 1.27, *p =* 0.138) [SES] Number of Children (>4 vs. ≤3) (Adjusted OR = 1.28, *p =* 0.057) [family characteristics]	
Werneck et al. (44)	**Significant:** No insurance (Adjusted OR = 4.87, *p =* 0.001) [SES] No family dentist (Adjusted OR = 3.96, *p =* 0.013) [SES] Frequency of snack consumption ≥2 per day (Adjusted OR = 3.79, *p =* 0.013) [feeding behavior]	
Schroth et al. (47)	Logistic Regression for ECC c/w age: **Significant:** Age of child (OR = 3.49, *p =* 0.0079) [age] **Not Significant:** Child brushes independently (OR = 0.59, *p =* 0.54) [oral hygiene behavior] Debris score (OR = 4.03, *p =* 0.17) [debris] Maternal rating of child's teeth (OR = 4.06, *p =* 0.11) [beliefs] Number of children in household (OR = 1.54, *p =* 0.10) [family characteristics] Logistic Regression for ECC w/out age: **Significant:** Maternal rating of child's teeth (OR = 7.49, *p =* 0.021) [beliefs] Number of children in household (OR = 1.94, *p =* 0.0057) [family characteristics] **Not Significant:** Child brushes independently (OR = 1.57, *p =* 0.52) [Oral hygiene behavior] Debris score (OR = 3.80, *p =* 0.12) [debris]	
Schroth et al. (49)	2nd Pilot Model: **Significant:** Children whose parents completed ≤Grade 12 were significantly more likely to have S-ECC (CI = 0.69, 3.778, OR = 9.3, *p =* 0.0046) [SES]	1st Pilot Model: **Significant:** PTH is significantly and independently associated with S-ECC (OR = 0.23, *p =* 0.004) Children with S-ECC significantly more likely to have higher PTH levels than caries free children **Not Significant:** 25(OH)D with S-ECC (OR = 2.2, *p =* 0.12). 2nd Pilot Model: **Not Significant:** 25(OH)D with S-ECC (OR = 1.7, *p =* 0.19).
Schroth et al. (22)		**Significant:** Children with S-ECC were nearly twice the odds of being classified as having low ferritin (OR = 1.48, *p =* 0.048). Children with S-ECC were over 6 times more likely to have IDA (OR = 6.58, *p =* < 0.0001).
Schroth et al. (23)	**Significant:** S-ECC related to lower household income (*p ≤* 0.001) [SES] S-ECC related to poorer rating of children's general health (*p =* 0.002) [beliefs]	**Significant:** S-ECC related to lower vitamin D levels (*p =* 0.04)
Schroth et al. (50)	**Significant:** ECC is related to infant age (≥14 months) at the time of dental examination (*p =* 0.01) [age] ECC is related to Enamel Hypoplasia (*p =* 0.001) [enamel hypoplasia] ECC is related to maternal prenatal 25(OH)D levels (*p =* 0.05) [prenatal nutrition]	
Schroth et al. (51)	**Significant:** Prevalence of ECC between the southern First Nation and Thompson (*p =* 0.00075) and the northern First Nation and Thompson (*p =* 0.00010) [community of residence] Prevalence of S-ECC between the southern First Nation and Thompson (*p =* 0.00081) and the northern First Nation and Thompson (*p =* 0.044) [community of residence] Decrease in S-ECC following community development (OR = 0.71, *p =* 0.026) [community of residence] Age (p < 0.05) **Not Significant:** Prevalence of ECC and S-ECC between the two urban centers (*p =* 0.13, *p =* 0.97) [community of residence]	
El Azrak et al. (11)	**Significant:** Age (months) with ECC (OR = 0.98, *p =* 0.034) [age] Debris Score with ECC (OR = 0.17, *p* < 0.001) and S-ECC (OR = 0.04, *p* < 0.001) [debris] Parent thinks child has dental problem with ECC (OR = 5.51, *p* < 0.001) and S-ECC (OR = 4.44, *p* < 0.001) [beliefs] Presence of enamel hypoplasia with ECC (OR = 6.10, *p =* 0.031) and S-ECC (OR = 3.93, *p =* 0.048) [enamel hypoplasia] **Not Significant:** Child sex with S-ECC (OR = 1.80, *p =* 0.14) [Sex] No dental insurance with S-ECC (OR = 1.12, *p =* 0.77) [SES] Child has visited dentist in Winnipeg with ECC (OR = 1.80, *p =* 0.14) and S-ECC (OR = 1.32, *p =* 0.5) [Dental History]	

*OR, Odds ratios; RR, Relative risk*.

Overall, the prevalence of ECC in the literature reviewed ranged between 0 and 98.9% (Table 1). Many of the identified publications involved preschool children from Manitoba (*n* = 16). As seen in Table 1, the prevalence of ECC appears to be high among Indigenous children, often affecting 85% of children or more. Prevalence was also considerably high among children from newcomer communities reaching as high as 79.5%.

Among the 27 studies reviewed that reported on the prevalence of ECC, seven also reported the prevalence of S-ECC. The average rates of S-ECC are lower than that of ECC, however, prevalence of S-ECC still varied greatly depending on the communities and participants involved (range: 21–95.8%). In one First Nation community, the prevalence of S-ECC among Indigenous children ranged from 81.9 to 95.8%. By contrast, the prevalence of S-ECC from several different communities including Hutterite, Indigenous, and immigrant or refugee families in southern Manitoba ranged as low as 21% to as high as 42.4%.

### Risk Factors

Childhood age is a recognized predictor of ECC and six of the reviewed Canadian studies considered it as a risk factor for ECC (Table 3). In 2007, a retrospective chart review to determine the prevalence of preschool dental decay reported that children who did not have their first dental visit before 24 months of age had increased odds of developing decay (40). These findings were further supported in a subsequent prospective study conducted by Schroth et al. (50) where they discovered that infants aged ≥14 months of age at the time of their first dental examination were significantly more likely to have ECC. Similarly, a 2010 study investigating the prevalence of ECC among Hutterite preschool children reported that childhood age was the strongest independent predictor of ECC (OR = 3.49) (47). More recently, Azrak et al. (11) found that older children had a mildly greater risk for developing ECC. Lastly, logistic regression analysis used in a serial cross-sectional study among four Manitoba communities revealed that childhood age was significantly related to S-ECC (Table 2) (51).

**Table 3:** Canadian studies using Logistic Regression to assess Caries risk factors.

**Risk factors**	**Number of studies assessing risk factor type**	**Number of studies reported significant associations with risk factor type**
Age	6	5 (83.3%)
Sex	5	1 (20%)
Socioeconomic Status (SES)	7	4 (57.1%)
Beliefs	4	4 (100%)
Family Characteristics	6	3 (50%)
Behavior	1	1 (100%)
Feeding Behavior	3	2 (66.7%)
Oral Hygiene Behavior	2	1 (50%)
Debris/Plaque	3	1 (33.3%)
Enamel hypoplasia	2	2 (100%)
Dental History	3	2 (66.7%)
Fluoride Exposure	2	1 (50%)
Community of Residence	2	1 (50%)
Ethnicity	1	1 (100%)
Prenatal Nutrition	1	1 (100%)

Another potential risk factor for caries development that was investigated among some of the Canadian studies was the sex of the child. However, out of five studies that employed logistic regression to investigate this relationship, only one 2007 study found males to be at 38% greater risk for developing ECC than females (Tables 2, 3) (40).

Three studies examined the dental history of children as a potential risk factor for caries development (Tables 2, 3). Children with a baseline decayed and filled surface (dfs) score of ≥5 were 4.88 times more likely to have caries over a 2 year interval vs. those whose dfs score was between 0 and 4 (45). In addition, one publication reported that children with a history of missed dental appointments had an 82% greater risk for developing ECC (OR = 1.82) (40). However, a more recent study did not find the location at which dental care was received to be a significant risk indicator for caries (11).

Three studies observed the relationship between debris score and ECC (Table 3). Two of these studies did not find the association to be statistically significant. However, one documented that the presence of debris on primary teeth was significantly and independently associated with ECC and S-ECC (11).

Enamel hypoplasia has been identified as additional risk factor in ECC development. Two studies reported that enamel hypoplasia was significantly and independently associated with ECC (Table 3). This was first verified in a prospective cohort study where they found that infants with enamel hypoplasia in the form of pits and missing enamel were significantly more likely to have ECC (50). Another study reported that children with enamel hypoplasia were 6.1 and 3.9 times more likely to have ECC and S-ECC, respectively (11).

Specific behavioral factors were categorized and examined for any relationships with ECC. One study investigated difficult child behaviors as a potential risk indicator for caries and found it to be significantly associated to the onset of ECC but not S-ECC (Table 2) (41). Three studies also considered how different feeding practices may contribute to ECC development; two of which were reported to be significant (Table 3) (10, 41, 44). Certain behaviors such as the bottle propping, the frequency of snack consumption, or having liquids in the mouth for prolonged periods of time were associated with ECC, however the results remain mixed. Lastly, behaviors related to child oral health practices were explored by two studies as possible risk indicators for ECC (Table 3). One reported that children who had difficulty brushing were significantly more likely to get ECC but not S-ECC (41). The other reported that independent brushing by the child did not contribute to the onset of ECC (47).

Four studies have considered the influence of caregiver beliefs and attitudes on the incidence of ECC in preschool children (Table 3). In 2007, Schroth et al. (38) found that children whose caregivers who believed it is safe to allow an infant to nurse throughout the night in bed were 45% less likely to experience decay in the form of caries(OR = 0.55). In addition, parents who believed that rotten teeth could impact their child's health were more than four times as likely to have children with ECC (OR = 4.33). However, whether parents agreed with the practice of bottle-feeding children after 1 year of age was not associated with ECC (Table 2) (38).

Certain socioeconomic factors such as low household income and the level of parental education or employment status can significantly increase the threat of caries development. Among these factors, two studies specifically assessed whether low annual household income is a risk indicator for both ECC and S-ECC (Table 2). In each instance, a lower household income increased the strength of an association with both forms of caries (23, 40). These findings were further supported in a cross-sectional study of 349 Inuit children that documented a higher family income as a significant protective factor against caries onset. Four studies also investigated the relationship between paternal education and employment status with presence of decay. Schroth and Cheba (40) did not find any significant differences between maternal employment status and deft scores. Similarly, no associations were observed between ECC and the mother's level of education when logistic regression was performed on participants from a rural Manitoban community (10). This finding was further supported by a RCT conducted with children in Ontario, where they did not find the level of parental education to be related to the child's development of ECC (OR = 1.27, *p* = 0.138) (45). These results were in sharp contrast to a subsequent pilot study investigating the relationship between vitamin D and S-ECC, which reported that children with parents whose educational attainment was ≤ grade 12 were 9.3 times more likely to have S-ECC (49).

Specific characteristics of the families children belonged to were also assessed for the presence of any significant associations with ECC (Table 3). Three studies considered the impact of family size on a child's likelihood for developing ECC. Neither Schroth and Moffatt (10) nor Lawrence et al. (45) found that children from larger families were at increased risk for developing ECC (Table 2). These findings were further supported by a pilot study of Hutterite children in southwest Manitoba (OR = 1.54, *p* = 0.10) (47). However, a subsequent regression model that controlled for childhood age did report statistical significance between family size and ECC (OR = 1.94) (47). One study reported that children coming from single parent households were more likely to be caries-free than those living with two parents (40).

The same pilot study of Hutterite preschool children did not find any significant association between the maternal rating (fair/poor/very poor) of their child's teeth and ECC (OR = 4.06, *p* = 0.17) (47). However, when age was excluded from regression analysis, low maternal ratings of childhood oral health were 7.49 times more likely to develop ECC (47). Since then, other studies have reported similar findings where lower parental ratings of their child's general or oral health was associated with a greater likelihood of experiencing caries (11, 23).

An in depth analysis on the prevalence of caries was conducted in preschool children under the care of the Children's Aid Society of Toronto (CAST), a not-for-profit agency that provides alternate care for young abused or neglected children. The results suggested that the period of time spent under CAST care did not significantly influence the risk of having S-ECC (OR = 1.29, *p* = 0.73) (43). However, they found that having permanent guardianship status of the children in care was protective against ECC development but not S-ECC (OR = 0.14, 0.22) (43). Conversely, an increase in the number of times children were admitted to CAST care was protective against the onset of S-ECC but not ECC (OR = 0.16, 0.42) (43).

One study investigated how differential access to dental care is related to ECC (44) (Table 2). This study was conducted on Portuguese-speaking immigrants in Toronto and revealed that children with ECC were more likely to be from families without reliable access to dental care and often lacking dental insurance.

Two studies analyzed the association between ECC and community of residence (urban/rural). One determined the prevalence of ECC among children accessing a community dental clinic and found that the prevalence of ECC among children from Winnipeg did not differ significantly from those residing in rural Manitoba (Table 2) (40). Alternatively, the other reported that the prevalence of ECC differed significantly between a southern First Nation and Thompson and a northern First Nation and Thompson, but not between the two urban centers investigated (51). In addition, significant differences in S-ECC were observed between the southern First Nation and Thompson, the northern First Nation and Thompson, but not between Winnipeg and Thompson (51).

Fluoride exposure or the lack thereof was investigated by several studies as a potential risk factor for ECC. A RCT reported that the scheduled application of fluoride varnish (FV) over a 2 year interval led to an 18.3% reduction in ECC among First Nation children and 24.5% reduction in the overall study population (45). Logistic regression further revealed that participants in the FV treatment group were 1.96 times less likely to have caries than comparable controls (45). By contrast, the presence of fluoridated water was not found to be protective against ECC or S-ECC development in another included study (41).

### Nutrition and Well-Being

Recently, more robust analysis involving logistic regression performed by our group has validated these initial findings of associations between ECC and nutritional health and well-being (Table 2). In 2013, a case-control study involving nearly 300 preschool children and documented that children with S-ECC were nearly twice the odds of having low ferritin and over six times more likely to have IDA than caries free children (22). The authors further extended their nutritional analysis to investigate the relationship between caries and vitamin D status. They reported that S-ECC was significantly associated with lower levels of vitamin D and elevated levels of parathyroid hormone (PTH) (23, 49). In 2014, a prospective study further supported the relationship between caries and vitamin D status as they found that low maternal prenatal vitamin D levels were associated with an increased risk of caries in infants (50).

### Microbiome

Only one study was identified that explored the composition of the oral microbiome of children with S-ECC (Table 1). This case control study involved First Nations and Métis children with and without ECC (54). The microbiome analysis of the 16S rRNA gene identified 10 phyla, 95 genera, and 290 species (54). The alpha (Within-sample) diversity analysis did not show any differences in species richness and phylogenetic diversity between groups (54). However, the beta diversity analysis revealed a significant cluster of samples according to caries-status (caries-free vs. S-ECC) (54). Taxonomic classification revealed that the levels of 28 species were significantly different between the groups (54). The caries-free group had 5 and 2-fold higher abundances of Streptococcus gondonii and Streptococcus sanguinis, respectively (54). Conversely, the S-ECC group had 7 and 9-fold higher levels of Haemophilus species (HOT 036) and Porphyromonas species (HOT 284) (54). Further, Veillonella species (HOT 780) was 4.6 times higher in the S-ECC group (54). Streptococcus mutans was detected in both groups, but was three times higher in the S-ECC group (54). These findings indicate that S-ECC is related to the structure of the plaque microbial communities.

## Discussion

This review reveals that there have been numerous investigations in Canada on ECC over the last two decades. Many of the studies involved populations often to be considered at high risk for caries, including Indigenous children, those from disadvantaged communities, and newcomer populations to Canada. Unfortunately, there have been no national representative samples to study the true prevalence of ECC in the Canadian population. In the absence of such data, we must rely on regional studies, review national surveys of parental reported ECC in specific populations, or look to pediatric dental surgery under general anesthesia to treat ECC as a proxy measure for the burden of disease in the infant and preschool population in Canada (3, 27, 28).

Despite all the advancements in preventive dentistry, ECC continues to be the most common reason for pediatric day surgery in Canadian hospitals and surgical centers (3). While the average national rate for surgery under general anesthesia to treat caries is 12.1/1,000 children, it is apparent that rates are considerably higher in certain regions of Canada, including rural and remote northern communities, where rates can exceed 100/1,000 children (3, 28). Children living in communities with a high proportion of Indigenous persons are nearly eight times more likely to undergo dental surgery for ECC while those living in rural regions are over three times as likely (3). In addition, children from the least affluent communities are 3.7 times more likely to undergo rehabilitative dental surgery than children from more affluent communities (3).

SES is a well-recognized determinant of oral health and was identified as a risk factor in several of the Canadian studies reviewed. Poverty, inability to pay for or lack of dental insurance can limit access to primary dental care and prevention and increase the risk for ECC and complex dental treatment (3, 4, 57). It can also prevent families from purchasing nutritious foods and oral hygiene supplies, which prevents parents from adopting dental friendly routines at home (8). Parental education and employment status are also some of the social determinants that influence caries development (58). Unfortunately, oral health is not part of Canada's universal healthcare system.

Some of the Canadian studies reviewed provide evidence that past oral health status and the presence of plaque are associated with ECC. Previous caries experience is actually the strongest predictor of future caries (59, 60). Unfortunately, there is considerable variability in what clinical assessments (e.g., plaque/debris levels, developmental defects of enamel) are made during examinations of preschool children in research studies. In fact, only two studies examined enamel hypoplasia as a risk factor, with both finding that these developmental defects of enamel are strongly associated with increased caries risk (11, 50). For many years, enamel hypoplasia has been an underappreciated risk factor for ECC, but there is increasing awareness of its contribution to the caries process as it is easily colonized by cariogenic microorganisms, which allows the caries process to progress more rapidly in these areas as either enamel is thinner or missing (50, 61–64). Future epidemiological studies into ECC should also collect clinical measures of enamel hypoplasia to understand its prevalence, role, and to identify those factors that are associated with its increased prevalence.

Infant feeding practices, such as bottle-feeding and breastfeeding, are complex processes that are difficult to investigate in typical studies on ECC (65). The lack of standardized questioning on these practices and the degree of breastfeeding (e.g., exclusive breastfeeding, partial breastfeeding, mixed feeding with expressed breastmilk), pose challenges as does the fact that such questioning can also be subject to recall bias. Many studies have not properly controlled for other potential modifiers such as the introduction of solids, other dietary behaviors, vitamin D supplementation, and the child's oral hygiene routine (65). While breastfeeding has sometimes been mistaken as a risk factor for ECC in the past, it is actually protective and associated with lower odds for caries (1, 27, 66, 67). However, breastfeeding beyond 24 months of age has been reported to be associated with an increased risk for ECC (68, 69).

Several Canadian publications have reported on the association between ECC and childhood nutritional health status and well-being and have added to our understanding of the oral-systemic relationship (22, 23, 49, 55, 56). The first Canadian report on the potential association between S-ECC and malnourishment suggested that many children with S-ECC suffered from low ferritin and met the definition for both iron deficiency (ID) and iron deficiency anemia (IDA) (21). Low maternal prenatal vitamin D concentrations was identified as a risk factor in one Canadian study (50). Since then, other investigators have also identified that vitamin D levels and prenatal vitamin D intake are associated with increased risk for ECC (70–72). In the few years it has been revealed that children with S-ECC are more likely to have lower serum levels of vitamin D, lower iron concentrations, and be both ID and have IDA than caries-free children (22, 23, 49). In many of these children these nutritional deficiencies actually co-exist (55). This provides more evidence that ECC is not just about teeth. Canadian children with S-ECC are also known to have higher BMI z-scores than caries-free counterparts. Current research is underway with our team to see whether nutritional status and well-being improve for these children following rehabilitative dental surgery under GA.

The advancement of modern microbiological techniques is undoubtedly enhancing our understanding behind the composition of the microbiome and risk for ECC. While only one Canadian study was identified, the field continues to grow (54, 73, 74). This will inevitably lead to future discussions on incorporating microbiological assessments into caries risk assessment (CRA) and risk prediction at the individual level (74, 75) Current research funded by the Canadian Institutes of health Research is underway to determine the role of taste genes and functional plaque microbiome in caries risk in young children in Manitoba Canada.

CRA is garnering increasing attention and is assisting in shifting our approach to managing caries from a restorative approach one that is non-restorative and minimally invasive. CRA can assists dental providers in tailoring clinical care decisions unique to the individual (76). It is vital that dental professionals familiarize themselves on how to undertake regular CRA of their young patients. CRA tools can also be used by non-dental primary healthcare professionals to screen children, determine their caries-risk, and provide prevention services, including fluoride varnish and anticipatory guidance. Several dental and pediatric organizations have developed CRA tools that can be used to help guide practitioners in determining someone's likelihood of developing caries. As parental belief and values can influence whether caries risk, it might be prudent to consider incorporating questions into CRA tools (38).

Early visits offer tremendous hope to curbing the problem of ECC. While there is growing awareness of the importance for such preventive dental practices, not all Canadian children are benefiting (77–80). Professional dental organizations, including the Canadian Dental Association call for the first dental visit no later than 12 months of age (81). In an attempt to increase dentist and public awareness, the Manitoba Dental Association established its Free First Visit program and the Canadian Dental Association introduced its First Visit First Tooth initiative (77–79).

As previously mentioned, many of the studies assessed in our review focused on Indigenous children, those from newcomer families, and those from disadvantaged communities, all who are known to have a higher burden of ECC than many other Canadian children. Different populations can have different risk factors for ECC as cultural practices and beliefs can play a role. However, one thing that is often common between our diverse at-risk populations is limited access to care and early childhood oral health promotion. Several programs and early childhood oral health initiatives are underway in regions of Canada. The federal Children's Oral Health Initiative is focused on improving the oral health status of young First Nations and Inuit children in Canada by emphasizing prevention and minimally invasive approaches to caries management over traditional restorative methods (82).

The Healthy Smile Happy Child (HSHC) initiative in Manitoba, Canada is a well-established, multidisciplinary partnership that works to address the problem of ECC while also considering the contributing health determinants (16, 51, 83, 84). HSHC is guided by three key pillars; (1) community engagement and development, (2) knowledge exchange, and (3) research, evaluation, and quality improvement (51, 85).

Key objectives of the HSHC initiative are to (1) promote the initiative and gain community awareness and acceptance of the importance of early childhood oral health, (2) use existing early childhood and family focused community-based programs, services and activities to deliver the oral health promotion and ECC prevention activities, and (3) recruit and train natural leaders, including service providers, to assist in program development and to deliver the ECC prevention program on an ongoing basis (51, 84). Further goals are to (4) scale-up capacity within existing programs and communities to assist in the sustainability of the promotional and educational program; and 5) determine the impact this would have on preschool oral health, parental knowledge, and attitudes regarding ECC, and knowledge of existing services and health care providers of the importance of prevention (51, 84). Evidence arising from the HSHC initiative's community development experiences over the past 18 years reveal that their approach is successful in improving parents' and caregivers' knowledge, attitudes, and behaviors toward early childhood oral health and results in significant reductions in caries scores and the prevalence of S-ECC over time (51, 83).

During our review of the Canadian ECC literature, it became apparent that there are some limitations with the Canadian literature. Many studies are cross-sectional in nature, with many being convenience samples of children and parents. A significant challenge with comparing risk factors between studies is the fact that investigators are not using a standardized set of questions or survey instruments. This means that different investigations into the same risk factors may yield differing findings even with the same population. The phrasing of questions into feeding and lifestyle behaviors are extremely variable. Further, not all studies are consistently looking at the risk factors. There is a lack of consistency in how questions about behaviors are being asked, which means that there is no uniformity in how risk factor variables are defined and measured between studies.

While there is finally consensus on how ECC and S-ECC are defined (1), there still is a lack of consensus of what is recorded as caries in epidemiological studies. The World Health Organization recommends that caries be diagnosed if pit and fissure or smooth surface lesions are cavitated and does not record incipient lesions as caries (86). This can result in an underestimation of the actual caries burden in high-risk children. While the recent Bangkok Declaration confirms the clinical definition of ECC, it does not speak to S-ECC (87, 88). However, S-ECC is still an important outcome to track in populations known to be at high risk for caries.

Going forward, researchers would be encouraged to try to use validated survey tools to help standardize data collection and analysis. Clinical researchers should also identify and record non-cavitated caries lesions. Lastly, researchers should continually strive to undertake more robust data analyses, such as multivariate regression (e.g., multiple logistic regression for ECC), to control for confounding variables.

## Conclusion

Current literature reveals that many Canadian children are affected by ECC. The development of ECC appears to be strongly associated with social determinants of health including low household income and the level of parental education or employment status. Associations were also observed between ECC and the child's age at first dental visit and parental beliefs about child's oral health. Children with enamel hypoplasia are also at significantly greater odds for experiencing caries. Future research should include assessments of developmental defects of enamel to better understand the association between enamel hypoplasia and ECC. Multiple logistic regression should become standard practice to identify true risk factors for ECC as this methodology can help to control for confounders.

## Author Contributions

AP and SS contributed to design, acquisition, analysis, and interpretation, drafted manuscript, critically revised the manuscript, gave final approval, and agrees to be accountable for all aspects of work ensuring integrity and accuracy. JL contributed to design, drafted manuscript, contributed to interpretation, critically revised the manuscript, gave final approval, and agrees to be accountable for all aspect of work ensuring integrity and accuracy. VC drafted manuscript, critically revised manuscript, gave final approval, and agrees to be accountable for all aspects of work ensuring integrity and accuracy. CG contributed to acquisition, critically revised manuscript for important intellectual content, gave final approval, and agrees to be accountable for all aspects of work ensuring integrity and accuracy. RS contributed to conception and design, contributed to acquisition, contributed to analysis, contributed to interpretation, drafted the manuscript, critically revised the manuscript, gave final approval, and agrees to be accountable for all aspect of work ensuring integrity and accuracy.

### Conflict of Interest

The authors declare that the research was conducted in the absence of any commercial or financial relationships that could be construed as a potential conflict of interest.
